# Periodontal Health and Oral Microbiota in Patients with Rheumatoid Arthritis

**DOI:** 10.3390/jcm8050630

**Published:** 2019-05-08

**Authors:** Kaja Eriksson, Guozhong Fei, Anna Lundmark, Daniel Benchimol, Linkiat Lee, Yue O. O. Hu, Anna Kats, Saedis Saevarsdottir, Anca Irinel Catrina, Björn Klinge, Anders F. Andersson, Lars Klareskog, Karin Lundberg, Leif Jansson, Tülay Yucel-Lindberg

**Affiliations:** 1 Department of Dental Medicine, Division of Periodontology, Karolinska Institutet, 14104 Huddinge, Sweden; anna.m.l.lundmark@gmail.com (A.L.); linkiatlee@gmail.com (L.L.); anna.kats@hotmail.com (A.K.); bjorn.klinge@ki.se (B.K.); leif.jansson@sll.se (L.J.); 2 Center for Rheumatology, Academic Specialist Center, Stockholm Health Services, 10235 Stockholm, Sweden; guozhong.fei@sll.se; 3 Department of Dental Medicine, Division of Orofacial Diagnostics and Surgery—Image and Functional Odontology, Karolinska Institutet, Huddinge 14104, Sweden; daniel.benchimol@ki.se; 4 Science for Life Laboratory School of Biotechnology, KTH Royal Institute of Technology, 17121 Stockholm, Sweden; yue.hu@ki.se (Y.O.O.H.); anders.andersson@scilifelab.se (A.F.A.); 5 Department of Microbiology, Tumor and Cell Biology, Centre for Translational Microbiome Research (CTMR), Karolinska Institutet, 17164 Stockholm, Sweden; 6 Department of Medicine, Rheumatology Unit, Karolinska University Hospital, Solna, 17176 Stockholm, Sweden; Saedis.Saevarsdottir@ki.se (S.S.); anca.catrina@ki.se (A.I.C.); Lars.Klareskog@ki.se (L.K.); Karin.Lundberg@ki.se (K.L.); 7 Department of Periodontology, Faculty of Odontology, Malmö University, 20506 Malmö, Sweden; 8 Department of Periodontology at Eastmaninstitutet, Stockholm County Council, 11382 Stockholm, Sweden

**Keywords:** rheumatoid arthritis, periodontitis, periodontal disease, anti-citrullinated protein autoantibodies, rheumatoid factor, smoking, medication, *Porphyromonas gingivalis*

## Abstract

This study aimed to investigate the periodontal health of patients with established rheumatoid arthritis (RA) in relation to oral microbiota, systemic and oral inflammatory mediators, and RA disease activity. Forty patients underwent full-mouth dental/periodontal and rheumatological examination, including collection of blood, saliva, gingival crevicular fluid (GCF) and subgingival plaque. Composition of plaque and saliva microbiota were analysed using 16S rRNA sequencing and levels of inflammatory mediators by multiplex-immunoassay. The majority of the patients (75%) had moderate or severe periodontitis and the rest had no/mild periodontitis. Anti-citrullinated protein antibody (ACPA) positivity was significantly more frequent in the moderate/severe periodontitis (86%) compared to the no/mild group (50%). No significance between groups was observed for RA disease duration or activity, or type of medication. Levels of sCD30/TNFRSF8, IFN-α2, IL-19, IL-26, MMP-1, gp130/sIL-6Rß, and sTNF-R1 were significantly higher in serum or GCF, and April/TNFSF13 was significantly higher in serum and saliva samples in moderate/severe periodontitis. The microbial composition in plaque also differed significantly between the two groups. In conclusion, the majority of RA patients had moderate/severe periodontitis and that this severe form of the disease was significantly associated with ACPA positivity, an altered subgingival microbial profile, and increased levels of systemic and oral inflammatory mediators.

## 1. Introduction

An increased risk of periodontitis has been reported in patients with rheumatoid arthritis (RA) as compared to healthy controls [1] and conversely, an association between chronic periodontal infection and risk of developing RA has been suggested [2,3]. The relationships between periodontitis and RA include similar pathological mechanisms of chronic inflammation and bone destruction [4,5,6,7], increased production of cytokines, prostaglandins and matrix-degrading enzymes, as well as shared risk factors where cigarette smoking is the most highlighted [2,4,5,6,8,9,10]. In periodontitis, the chronic inflammation, which results in the destruction of tooth-supporting structures, is initiated by periodontal pathogens such as *Porphyromonas gingivalis* and a dysbiotic microbial community surrounding the periodontium [11].

The underlying etiological processes in RA are not fully understood, although ever since the identification of antibodies to citrullinated protein antigens (ACPAs) as specific markers predictive for the development of RA, associated also with disease severity and joint destruction [12], increased attention has been given to the etiological mechanisms of ACPA production. These antibodies are directed against post-translationally modified proteins containing the amino acid citrulline generated by the enzyme peptidyl arginine deiminase (PAD) during the process of citrullination [12,13]. Some recent studies suggest that immunity against citrullinated proteins may be initiated at a mucosal site (e.g., the lung or gingiva), and others point to a cross-reactivity scenario between microbial amino acid sequences and citrullinated self-proteins resulting in ACPA production [11,14,15]. The periodontal pathogens *P. gingivalis* and *Aggregatibacter actinomycetemcomitans* have been suggested to be involved in the generation of citrullinated antigens and the subsequent production of ACPA. Interestingly, *A. actinomycetemcomitans* was reported to induce hypercitrullination in host neutrophils, with hypercitrullination patterns similar to those observed in synovial fluid of RA patients [16]. *P. gingivalis*, on the other hand, has for some time been implicated in RA autoimmunity because of its unique ability to express a microbial PAD enzyme (*P. gingivalis* PAD, PPAD), which has the ability to citrullinate proteins, similar to the human PAD enzymes [2,17]. By citrullinating proteins in the periodontium, PPAD could trigger the production of ACPAs, which through epitope spreading may cross-react with citrullinated proteins in the joints resulting in a chronic inflammation and eventually joint destruction [2].

A relationship between periodontitis and RA has recently been supported by a systematic review and meta-analysis [1]. One of the largest studies that report an association (OR = 1.16; 95% CI: 1.12–1.20) between these two diseases was based on a register study including 13,779 Taiwanese patients with newly diagnosed RA and 137,790 controls [18]. A significant association between RA and periodontitis (OR = 1.17; 95% CI: 1.15–1.19, *p* < 0.001) was also reported in a Korean population based registry study comprising 57,024 patients with RA out of which 26,320 had periodontitis [19]. However, none of these studies were able to account for smoking, which is an important risk factor for periodontitis, as well as RA, and the studies lacked information about autoantibody status. Thus, the strength of the relationship between periodontitis and RA is still an area of interest for researchers and clinicians, as several studies have also failed to report an association between these two diseases. For example, in the largest prospective study conducted to date where 81,132 female nurses (292 RA and 80,840 controls) were followed for more than 12 years, no association was found between RA and periodontal surgery and/or tooth loss [20]. Likewise, in our recently published study of 6682 Swedish patients with established RA and matched healthy controls included in the Epidemiological Investigation of Rheumatoid Arthritis (EIRA) registry, we reported no increased prevalence of periodontitis diagnosis in patients with RA as compared to controls, and no differences in periodontitis prevalence based on ACPA or rheumatoid factor (RF) status [21]. Importantly, however, in that study, we were not able to assess the severity of the periodontal diagnosis in patients with RA using clinical parameters of periodontal disease. The aim of this study was, therefore, to investigate the severity of periodontitis (defined as severe, moderate or no/mild) [22] in Swedish patients fulfilling the 2010 American College of Rheumatology (ACR) criteria for RA in relation to autoantibody status (ACPA and RF), inflammatory mediators, RA disease activity and medication as well as the microbiota in saliva and subgingival plaque.

## 2. Experimental Section

### 2.1. Study Population

A total of forty-five volunteers (age 29 to 80) with chronic arthritis (mean disease duration 11 years) were recruited from two Rheumatology clinics at Karolinska University Hospital in Solna and Huddinge (Stockholm, Sweden) between January 2016 and January 2017. Five participants were excluded from the study due to not fulfilling the inclusion criteria for RA (the 2010 ACR Criteria for RA) [23], or having other types of chronic arthritis. The recruited participants underwent a full mouth dental and periodontal examination (third molars not included), performed by a single dentist (KE) calibrated by a periodontist (LJ). The examiner (KE) did not have information about the patients periodontal or rheumatological measurements (clinical or laboratory) beforehand. Based on the results from the dental examinations, the patients were divided according to their periodontal status, following the standardised clinical definition of periodontitis (intended for use in population-based studies) established by the Centers for Disease Control and Prevention and the American Academy of Periodontology [22]. All patients completed a health screen questionnaire including information about comorbidities, medication, smoking and alcohol habits, body mass index (BMI) as well as questions regarding education and place of birth. The participants had not received any periodontal treatment for at least 3 months prior to the dental examination. Exclusion criteria included pregnancy, lactation, other forms of arthritis as well as the use of antibiotics the last 3 months prior to examination. This study was approved by the Regional Ethical Review Board in Stockholm (Dnr 2009/792-31/4 and 2015/766-32) and a written informed consent was obtained from all participants.

### 2.2. Clinical Assessments

The RA disease activity was assessed by using the DAS28 CRP (Disease Activity Score in 28-joints) [24] measuring total tender- and swollen- joint count (ranging from 0–28 joints), C-reactive protein (CRP, mg/dL), as well as the patient’s global assessment of health on a 10 cm visual analogue scale. A DAS28 score of >5.1 was considered high disease activity, whereas <3.2 equaled low disease activity and ≤2.6 reflected remission [25]. Patients’ self-reported functional status was measured using the Health Assessment Questionnaire (HAQ) [26], where functional ability was assessed by addressing eight general component categories (reach, grip, hygiene, dressing and grooming, eating, arising, walking and common daily activities). In the HAQ disability index, each question was scored from 0 to 3, corresponding to “without any difficulty” (score 0), “with some difficulty” (score 1), “with much difficulty” (score 2) and “unable to do” (score 3).

The periodontal condition was assessed by probing pocket depth (PPD), clinical attachment level (CAL) and bleeding index (BI) determined at 6 sites per tooth, and the presence of supragingival plaque (PI) at 4 sites per tooth. Stimulated salivary flow rate, number of missing and mobile teeth as well as the number of multirooted teeth with furcation involvement were also recorded. Periodontitis, defined by following international consensus criteria, was based on the interproximal measurements of CAL and PPD, excluding the third molars [22]. The severity of the disease was defined as severe (corresponding to ≥2 interproximal sites with CAL ≥ 6 mm, not on the same tooth, and ≥1 interproximal sites with PPD ≥ 5 mm), moderate (corresponding to ≥2 interproximal sites with CAL ≥ 4 mm, not on the same tooth, or ≥2 interproximal sites with PPD ≥ 5 mm) or no/mild (corresponding to neither severe nor moderate criteria) [22]. In addition to the periodontal status, the examination also included assessment of soft tissue pathologies and the number of decayed, missing and filled permanent teeth and tooth surfaces (DMFT/DMFS) describing the amount of dental caries, where the DMFT can range from 0 to 28 and the DMFS from 0 to 128 [27].

### 2.3. Collection and Preparation of Gingival Crevicular Fluid, Plaque, Saliva and Blood Samples

Gingival crevicular fluid (GCF) was collected by inserting two paper strips (Periopaper, Proflow Inc., Amityville, NY, USA) until slight resistance, at both the mesiobuccal and the distobuccal sites of the teeth with the deepest pockets. The paper strips were left within the gingival crevice for 30 s, pooled together and frozen at −80 °C until processing [28].

Subgingival plaque samples were collected from the deepest pockets by inserting four sterile paper points (Nordenta, Enköping, Sweden), two at the distolingual and two at the mesiallingual sites of the tooth, and left for 20 s [29]. Before the paper points were inserted supragingival plaque was removed by using cotton pellets and the tooth surface was dried with air. The samples from the same tooth were pooled together and frozen at −80 °C awaiting analysis.

Stimulated saliva samples were collected by using paraffin wax (1g, Ivoclar Vivadent, Liechtenstein), which the participants were instructed to chew on for a duration of 2 min. The volume of collected stimulated saliva was determined, the salivary flow rate recorded and the samples kept on ice until processing. Saliva samples were then centrifuged at 500× *g* for 10 min at 5 °C and supernatants collected and stored at −80 °C until analysis [28].

Blood samples were collected in BD Vacutainer SST tubes (MediCarrier AB, Stockholm, Sweden). Tubes were left standing at room temperature for at least 30 min before storing, in order to remove cells and clotting factors by allowing a clot to form. The samples were then centrifuged at 200× *g* for 10 min at 20 °C and the serum stored at −80 °C until analysis. The preparation of the samples is described in the Supplementary Methods (Preparation of samples).

### 2.4. Immunoassay Analysis

Serological markers of RA (ACPA and RF antibody status) were analysed using a multiplex immunoassay (Bio-Plex^®^ 2200 system, Bio-Rad, Hercules, CA, USA) for ACPA and nephelometry for RF (Karolinska University Hospital Laboratory, Sweden). Results were interpreted as ACPA-positive/RF-positive following the cut-off values for seropositivity (3 E/mL for ACPA and 20 E/mL for RF).

Levels of CRP in serum (measured between intervals 0.2–380 mg/L) were analysed at Karolinska University Hospital Laboratory using a near-infrared particle immunoassay (NIPIA) method and Beckman reagents on Synchron LX20 automated equipment (Beckman Coulter, Fullerton, CA, USA) [30]. Concentrations of CRP in saliva and GCF samples were analysed via commercially available ELISA kit (USCN Life Science, Wuhan, China), according to the manufacturer’s instructions. Briefly, PBS-diluted saliva (diluted 1:3) and GCF samples (diluted 1:2) were incubated with biotinylated CRP antibodies, followed by incubation with horseradish peroxidase conjugate. CRP-antibody levels were detected with tetramethylbenzidine reagent and the concentrations expressed as pg/ml. The detection limit of the CRP ELISA was 19.2 pg/ml with <12% inter-assay and <10% intra-assay variation.

Immunoassay kits (37-Plex inflammation panel, Bio-Rad, Hercules, CA, USA) were used to investigate the cytokine profile in serum (diluted 1:4), saliva and GCF (undiluted), according to manufacturer’s protocol. The analysed cytokines (sensitivities in pg/ml, in brackets) were APRIL/TNFSF13 (190), BAFF/TNFSF13B (34.7), sCD30/TNFRSF8 (1.0), sCD163 (16.8), Chitinase 3-like 1 (10.3), gp130/sIL-6Rβ (16.9), IFN-α2 (0.7), sIL-6Rα (1.5), IL-8 (2.7), IL-10 (0.6), IL-11 (0.05), IL-12 (p70) (0.1), IL-19 (0.2), IL-20 (3.6), IL-22 (1.1), IL-26 (1.2), IL-27 (p28) (0.1), IL-29/IFN-λ1 (1.6), IL-32 (12.3), IL-34 (51.9), IL-35 (3.7), LIGHT/TNFSF14 (10.2), MMP-1 (33.7), MMP-2 (39.7), MMP-3 (28.5), Osteocalcin (23.4), Osteopontin (91.3), Pentraxin-3 (0.8), sTNF-R1 (0.2), sTNF-R2 (3.2), TSLP (0.8) and TWEAK/TNFSF12 (0.5). The mean concentrations were expressed as pg/ml and the inflammatory mediators that were below the detection limit were excluded.

### 2.5. 16S rRNA Sequencing and Quantitative Polymerase Chain Reaction (qPCR)

The amount of bacterial DNA from saliva and subgingival plaque samples of the 40 included patients was determined by using the 7500 Fast Real-Time qPCR System (Applied Biosystems, Foster City, CA, USA). Ten samples with less than in total 0.5 ng/µL DNA were excluded from qPCR analysis, whereas 3 samples with less than 0.5 ng/µL DNA were excluded from 16S rRNA gene sequencing. For the preparation of the 16S rRNA amplicon libraries, 2 ng genomic DNA (gDNA) from each saliva and plaque sample were used as template to amplify the V3-V4 regions of the 16S rRNA gene, with final concentrations for PCR reactions being 1X KAPA HotStart ReadyMix (KAPA Biosystems, Wilmington, MA, USA), 1.0 µM 341′F primer (CCTAHGGGRBGCAGCAG), 1.0 µM 805R primer (GACTACHVGGGTATCTAATCC) [31,32] and 0.1 ng/µL gDNA. The PCR during amplification was programmed as follows, initial incubation at 98 °C for 2 min, 26 cycles of 98 °C for 20 s, 54 °C for 20 s, 72 °C for 15 s, followed by an elongation step of 72 °C for 2 min. The samples were then purified with Polyethylene Glycol 6000 (Merck Millipore, Darmstadt, Germany) and carboxylic acid beads (Dynabeads^®^ MyOne™, Thermo Fisher Scientific, Waltham, MA, USA) [33]. For indexing the sample amplicons, 12 µL of the purified product, 0.4 µM forward and reverse indexing primers and KAPA HotStart ReadyMix (KAPA Biosystems, Wilmington, MA, USA) were mixed for PCR amplification. The indexing step followed the PCR programme: 98 °C for 2 min, 10 cycles of 98 °C for 20 s, 62 °C for 30 s, 72 °C for 30 s and a final elongation step of 72 °C for 2 min. Equimolar amounts of indexed samples were mixed and sequenced on the Illumina MiSeq platform (Illumina Inc, San Diego, CA, USA) at NGI/SciLifeLab Stockholm. Three samples were excluded due to their low number of reads (cutoff 10,000 reads). The median depth of sequencing, after exclusion of low-depth libraries, was 188,600 reads per sample (IQR: 166,669–209,905 reads).

For the qPCR analysis of subgingival plaque samples, two sets of primers were used for bacteria detection, one species-specific pair for *P. gingivalis* and the other pair targeting the bacterial 16S rRNA gene [34,35]. The qPCR analyses were performed as described in the Supplementary Methods (Quantitative Polymerase Chain Reaction, qPCR).

### 2.6. Sequence Count Data Processing and Analysis

To process the sequencing data the DADA2 pipeline [36] was used. Amplicon reads with low quality and primers were trimmed and filtered. The remaining reads were then dereplicated, the sequence variants were inferred, and the paired-end reads were merged by requiring 30 bp overlap with no mismatches. Results of two sequencing runs were then merged and a sequence table was constructed, followed by removal of chimeras. The reads were taxonomically assigned using RDP training set 14 [37]. A phylogenetic tree was constructed using the scripts align_seqs.py, filter_alignment.py, and make_phylogeny.py, as provided by QIIME [38]. The sequence table, taxonomic assignment, and phylogenetic tree were used to create a phyloseq object [39], which was used in all subsequent analyses.

In total 6538 taxa were identified from the merged sequencing table. The final dataset included 1090 taxa, after removing the sequence variants that only present in 5% or less of the samples. The analysis of differential abundance of microbes, reported as genus species (*sp*.), between saliva samples and plaque samples as well as between samples from RA patients with no/mild and moderate/sever periodontitis was performed using DESeq2 [40]. All statistical analyses were conducted in R, version 3.3.3 (R Core Team 2015. R Foundation for Statistical Computing, Vienna, Austria).

### 2.7. Statistical Analysis

The characteristics of patients with moderate/severe periodontitis were compared to the subjects with no/mild periodontal disease. Mann–Whitney U test or independent two-sample t-test was performed for comparing ordinal and continuous variables. For dichotomous variables, Chi-square test or Fisher’s exact test was used. The association between ACPA status, periodontitis severity and periodontal bacteria, estimated as odds ratio (OR) with 95% Confidence Interval (CI), was analysed using logistic regression analysis and adjusted for gender, age and smoking habits. All analyses were performed using the IBM SPSS Statistics 21.0 (IBM Corp., Armonk, NY, USA) package. Statistical significance was determined at *p* value < 0.05.

## 3. Results

### 3.1. Characteristics of the Study Population

The characteristics of the RA study population are summarised in Table 1. The group consisted of 40 patients fulfilling the ACR criteria for RA. The mean age of the total study population was 60 ± 11 years and the majority of the participants were women (88%). With regard to the periodontal health, the majority, 75%, of the included subjects had moderate/severe periodontitis (*n* = 30), with the rest (*n* = 10) having no/mild periodontal disease. Based on periodontal diagnosis, there were no significant differences between the two groups with regard to gender, BMI, RA disease duration, place of birth, alcohol consumption or the level of education. The groups were also comparable with respect to comorbidities and medications. Subjects with moderate/severe periodontitis were significantly (*p* = 0.010) older (mean age 64 ± 7.8 years) compared to those with no/mild periodontal disease (mean age 50 ± 14 years). With regard to smoking, there were significantly (*p* = 0.014) more never smokers in the group with no/mild disease as compared to the group with moderate/severe periodontitis (60% and 17%, respectively).

### 3.2. Periodontal and Dental Characteristics in Relation to Periodontitis Severity

During the periodontal and dental examinations, variables such as PI, BI, PPD, CAL, number of missing teeth, mobile and furcation involved teeth, DMFT/DMFS as well as stimulated salivary flow rate were recorded. The subjects with moderate/severe periodontitis had significantly higher (*p* values demonstrated in Supplementary Table S1) frequencies of most of the investigated variables, including PPD ≥ 4 mm, interproximal sites with PPD ≥ 5 mm, sites with CAL 1–2 mm, 3–4 mm or ≥ 5 mm, interproximal sites with CAL ≥ 4 mm or ≥ 6 mm, as well as significantly higher numbers of missing, mobile and furcation involved teeth, as compared to patients with no/mild periodontal disease (Supplementary Table S1). The group with no/mild periodontitis had no interproximal sites with PPD ≥ 5 mm or CAL ≥6 mm. Moreover, subjects with no/mild periodontitis had significantly less DMFT and DMFS as compared to those with moderate/severe disease. There were, however, no significant differences in PI or BI between the two groups, and also no differences in salivary flow rate (Supplementary Table S1).

### 3.3. Rheumatological Characteristics in Relation to Periodontal Status

The periodontal status in relation to rheumatological characteristics was also investigated. The majority (75%) of the study participants were ACPA-positive. When dividing the patients with regard to periodontitis severity, ACPA positivity was significantly (*p* = 0.032) more frequent in patients with moderate/severe periodontitis (86%), compared to the group with no/mild disease (50%) (Table 2). In addition, RF positivity showed a similar trend, although did not reach statistical significance. There were no significant differences between no/mild and moderate/severe periodontitis with regard to self-reported HAQ score, the DAS28 score reported by the patients rheumatologists or the CRP-levels in serum, saliva or GCF. With regard to RA disease activity, most of the participants had moderate disease activity (5 in the no/mild and 9 in moderate/severe group) or were in remission (4 and 10, respectively), irrespective of periodontal status (Table 2).

In the next series of studies, the participants were also analysed with regard to ACPA status. The ACPA-positive patients were taking significantly (*p* = 0.040) more non-steroidal anti-inflammatory drugs (NSAIDs) compared to the ACPA-negative subjects with RA (37% versus 0%, respectively) (Supplementary Table S2). There were, however, no differences in other medications (analgesics, DMARDs, biological DMARDs, glucocorticoids or bisphosphonates) based on ACPA status. Moreover, in the ACPA-positive group there were significantly (*p* = 0.038) more ex-smokers, as compared to the ACPA-negative group. In contrast, the ACPA-negative participants were mostly never smokers (*p* = 0.032). There were no significant differences in gender, age, BMI, RA disease duration, comorbidities, alcohol consumption, education or place of birth based on ACPA status (Supplementary Table S2). Moreover, based on ACPA status, there were no significant differences between the groups regarding plaque index (ACPA-positive 51% ± 19%; ACPA-negative 40% ± 18%) or bleeding index (ACPA-positive 32% ± 22%; ACPA-negative 41% ± 14%).

### 3.4. Levels of Inflammatory Mediators in Serum, Saliva and GCF Samples

**Figure 1:**
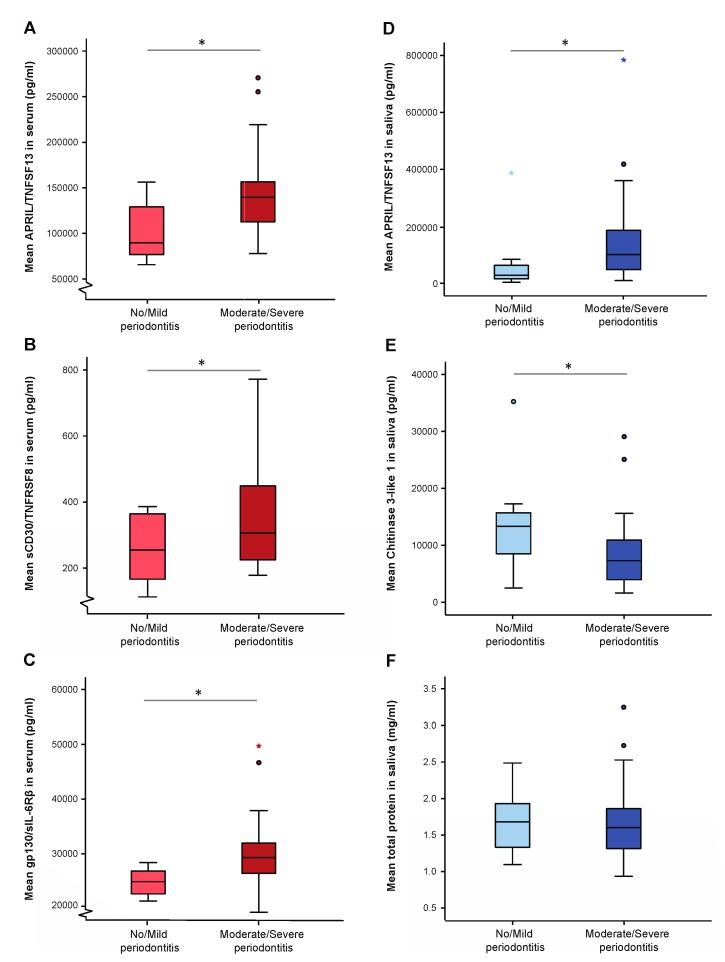
Levels of inflammatory mediators in serum and saliva samples. Mean (±SD) serum concentrations of APRIL/TNFSF13 (**A**), sCD30/TNFRSF8 (**B**) and gp130/sIL-6Rß (**C**); mean (±SD) saliva concentrations of APRIL/TNFSF13 (**D**), Chitinase 3-like 1 (**E**) and levels of total protein in saliva (**F**) from RA patients with no/mild and moderate/severe periodontitis. The differences between the groups were analysed by Mann–Whitney U-test. * *p* value < 0.05 was considered statistically significant. Circle indicates outlier and star indicates far outlier.

The levels of inflammatory mediators were analysed in serum, saliva and GCF samples from RA patients with no/mild and moderate/severe periodontitis (Figure 1 and Table 3). In serum, RA subjects with moderate/severe periodontitis had significantly higher levels of APRIL (TNFSF13) (*p* = 0.013), sCD30 (TNFRSF8) (*p* = 0.048) and gp130 (sIL-6Rß) (*p* = 0.01) compared to patients with no/mild form of periodontitis (Figure 1A–C). Similarly, patients with moderate/severe periodontitis had significantly higher salivary levels of APRIL (*p* = 0.048) compared to those no/mild periodontal disease (Figure 1D). In contrast, the salivary levels of Chitinase 3-like 1 were significantly (*p* = 0.041) lower in patients with moderate/severe periodontitis compared to no/mild periodontitis group (Figure 1E). In GCF samples, significantly (*p* < 0.05) higher levels of IFN-α2, IL-19, IL-26, MMP-1 and sTNF-R1 were observed in RA patients with moderate/severe periodontitis compared to corresponding RA subjects with no/mild periodontitis (Table 3). However, there were no significant differences in total protein concentrations determined in saliva or GCF samples (Figure 1F and Table 3, respectively) between the two groups.

### 3.5. Microbial Profile of Subgingival Plaque and Saliva Samples

**Figure 2:**
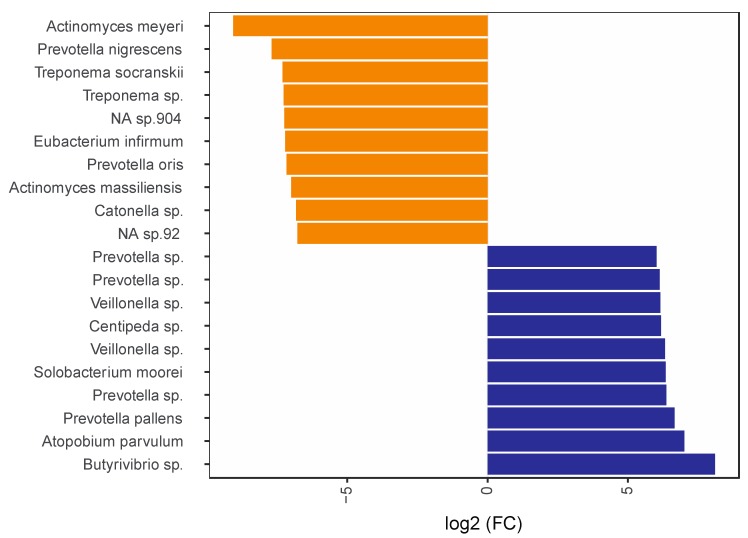
Most highly differentially abundant bacterial species in plaque compared to saliva samples. The graph demonstrates log2 Fold Change (FC) for the ten most enriched as well as the ten most depleted bacteria in plaque compared to saliva from patients with RA irrespective of periodontitis. Negative log2 FC indicates higher abundance in plaque samples (orange bars) and positive values indicate higher abundances in saliva samples (blue bars). NA, genome reference not available. The differences between the groups were analysed using DESeq2 [40]. *Adjusted *p* value < 0.05 was considered statistically significant.

**Figure 3:**
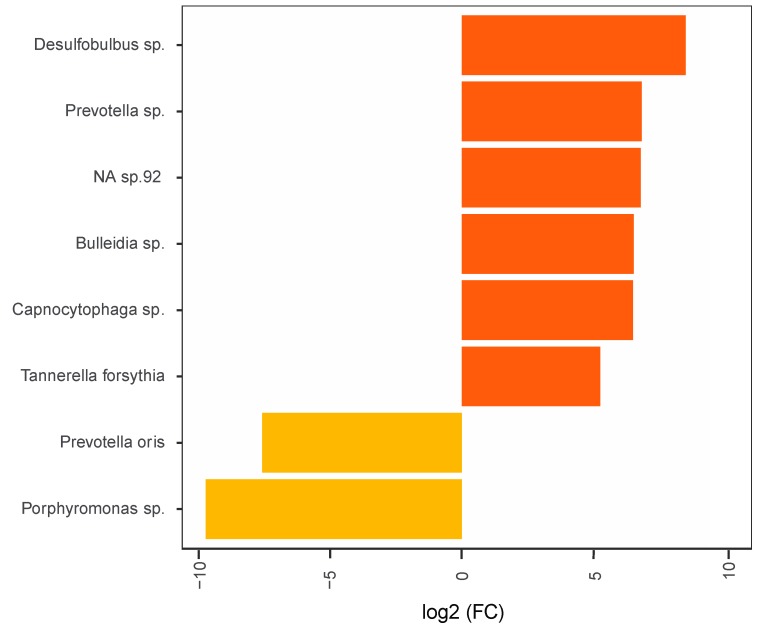
Most highly differentially abundant bacteria in plaque from patients with RA in relation to periodontitis severity. The graph demonstrates log2 Fold Change (FC) for the most highly enriched and depleted bacteria in plaque from RA patients with no/mild and moderate/severe periodontitis. Negative log2 FC indicate higher abundance in patients with no/mild periodontitis (light orange bars) and positive values indicate higher abundances in those with moderate/severe periodontitis (dark orange bars). NA, genome reference not available. The differences between the groups were analysed using DESeq2 [40]. *Adjusted *p* value < 0.05 was considered statistically significant.

The oral microbial profiles of the RA patients were analysed by 16S rRNA gene sequencing plaque and saliva samples. The most highly differentially abundant microbes in plaque samples compared to saliva samples, irrespective of periodontal status, are demonstrated in Figure 2. The ten most significantly (*p* > 0.05) enriched microbes detected in plaque compared to saliva were *Actinomyces meyeri*, *Prevotella nigrescens*, *Treponema socranskii*, *Treponema* sp., *Eubacterium infirmum*, *Prevotella oris*, *Actinomyces massiliensis*, *Catonella* sp. and two non-identified species (*NA* spp.). In contrast, in saliva samples the most enriched microbes included *Butyrivibrio* sp., *Atopobium parvulum*, *Prevotella pallens*, *Solobacterium moorei*, *Centipeda* sp., two *Veillonella* spp., as well as three *Prevotella* spp. (Figure 2). The oral microbial profiles of patients with RA were also investigated in relation to periodontal status (Figure 3). The results showed that patients with moderate/severe periodontitis had significantly (*p* < 0.05) higher abundance of *Desulfobulbus* sp., *Prevotella* sp., *Bulleidia* sp., *Capnocytophaga* sp., *Tannerella forsythia* and a *NA* sp. in plaque, when compared to RA with no/mild periodontitis. In contrast, *Prevotella oris* and a *Porphyromonas* sp. were more abundant in patients with no or mild periodontitis (Figure 3). In saliva, there were no significant differences in the detected bacterial species based on periodontal diagnosis (Supplementary Table S3).

Subgingival plaque samples of patients were also analysed for the presence and quantity of *P. gingivalis* using qPCR. The results showed that *P. gingivalis* was present in higher degree (62%) in the moderate/severe periodontitis group compared to no/mild periodontitis group (50%), although the difference was not significant (Supplementary Table S4). 

## 4. Discussion

The objective of this study was to investigate the severity of periodontitis in relation to RA-associated clinical and immunological parameters, medication and comorbidities in patients with established RA. In this well-defined cohort, our results show that moderate/severe periodontitis is common in patients with RA, especially in ACPA-positive RA. In addition, we identified significantly enriched/differentially abundant bacteria in subgingival plaque of RA patients with moderate/severe periodontitis compared to those with no or mild periodontal disease. Moreover, the levels of both systemic and oral (saliva and GCF) inflammatory mediators were significantly higher in RA with moderate/severe periodontitis. Notably, the proliferation-inducing ligand APRIL (TNFSF13), a member of the TNF-receptor superfamily, was significantly increased in serum and saliva samples.

ACPAs are important diagnostic and prognostic serological markers in RA, and their presence is known to predict the development of RA [41]. In the current study, our results showed that ACPA positivity was significantly more common in RA patients with moderate/severe periodontitis when compared to those with no/mild form of disease. In agreement with our results, a more severe form of periodontitis (indicated by higher percentage of sites with alveolar bone loss) was reported in ACPA-positive patients with RA, in an American cohort [42]. Conversely, in a recent study, ACPA positivity only correlated with the gingival inflammation markers bleeding on probing and gingival index, but not with the severity of periodontitis assessed by CAL or PPD [43]. In our study, we found no differences in oral hygiene or gingival inflammation based on ACPA status. One possible explanation for this might be that the ACPA-positive patients in our cohort were taking significantly more NSAIDs, which may reduce and mask the signs of gingival inflammation [44].

Using 16S rRNA gene sequencing, previous studies have investigated the subgingival microbiome of patients with RA and compared the results of subjects with osteoarthritis and healthy controls without periodontitis [45,46]. These studies did neither report subgingival profiles/compositions discriminating between RA and osteoarthritis [45], nor did they demonstrate differences between RA and controls without periodontitis with regard to the periodontal pathogens *P. gingivalis* or *A. actinomycetemcomitans* [46]. In our study, investigating the oral microbial composition in RA based on periodontitis severity, the subgingival microbial profile differed significantly between patients with moderate/severe and no/mild periodontitis. The species observed in higher abundance in moderate/severe periodontitis were *Desulfobulbus* sp., *Prevotella* sp., *NA* sp., *Bulleidia* sp., *Capnocytophaga* sp. and *Tannerella forsythia*, and two were more abundant in no/mild periodontitis (*Prevotella oris* and a *Porphyromonas* sp.). Similarly, in Norwegian RA cohort, the microbial composition of subgingival plaque also differed in relation to periodontal severity measures (such as gingival bleeding and PPD) [47]. However, in that study, RA disease status and medication (glucocorticosteroids) were found to be associated with different microbiome composition [47], in contrast to our study where no differences were found between no/mild and moderate/severe periodontitis group in terms of these potential confounding factors, therefore likely not affecting our results. Moreover, the prevalence of the periodontal pathogen *P. gingivalis*, assessed in 50 - 62% of the patients in our study, is in line with previous reports showing that this bacterium was present in 47% of patients with established RA and in 55% of newly-diagnosed RA patients [48]. We did not observe any significant differences in the prevalence of *P. gingivalis* in subgingival plaque based on the severity of periodontitis. In a previous study by Scher et al., *P. gingivalis* was reported to be more prevalent in newly-diagnosed treatment naïve RA patients with advanced periodontitis as compared to a corresponding group of RA without periodontitis [48]. This discrepancy could potentially be explained by the use of RA-medications, supported by the finding that DMARDs may modulate the microbiome in patients with RA [49]. It has been hypothesised that microbiome changes are partly driven by systemic inflammation and can be modulated by DMARDs, abating the inflammatory response [50]. Even though there were no differences in type of medications used between the groups in our study, we did not investigate the use of specific subgroups of DMARDs or biological DMARDs. Moreover, as proposed by Zhang et al., the differences in modulation of the oral microbiome could also be due to differences among RA patients [49], or possibly differences in response to RA-medication, which may explain why prevalence of *P. gingivalis* did not differ between RA patients with no/mild and moderate/severe periodontitis despite similar use of DMARDs therapy in the groups.

Inflammatory mediators play a central role in the pathogenesis of chronic inflammatory diseases such as periodontitis and RA, promoting autoimmunity and maintaining a chronic inflammation that collectively contribute to tissue and bone destruction [5,51]. Persistently increased levels of various pro-inflammatory cytokines (e.g., TNF-α and IL-6) have been well documented in RA and periodontitis, and several cytokine-targeting therapies are successfully used in RA treatment [52,53]. In the present study, the levels of sCD30 (TNFRSF8), sTNF-R1, gp130 (sIL-6Rß), IL-19, IL-26 and MMP-1 were increased in serum, saliva or GCF samples from subjects with moderate/severe periodontitis as compared to no/mild disease. Interestingly, RA patients with moderate/severe periodontitis also had increased levels of APRIL both in serum and in saliva samples. APRIL, also known as TNFSF13, is a proliferation ligand and a member of the TNF-receptor superfamily [54]. This cytokine has together with BAFF an important role in the survival and maturation of B-cells [54], and could therefore potentially be important for the production of antibodies in RA. The overexpression of APRIL in serum has been reported in several autoimmune diseases including RA [54], and increased expression of APRIL mRNA has also been detected in gingival tissue from patients with periodontitis when compared to non-periodontitis controls [55]. However, to our knowledge this is the first study reporting significantly different levels of APRIL both in serum and saliva samples of RA patients with different degrees of periodontal disease. Thus, given that overexpression of APRIL/BAFF is suggested to contribute to the autoimmune diseases [54,56], this system may be a key mediator involved in the link between RA and periodontitis. Only one protein, Chitinase 3-Like-1 (also known as YKL-40), was significantly reduced in saliva of patients with moderate/severe periodontitis. Although, this protein is a potential inflammatory biomarker in arthritis, its role as a marker for disease diagnosis is debated [57]. In RA, circulating levels of Chitinase 3-Like-1 reflect cartilage degradation and synovial inflammation [57,58], and in osteoarthritis this cartilage glycoprotein have been suggested as a potential target for treatment [59]. To our knowledge, this is the first study reporting the levels of Chitinase 3-Like-1 in saliva, both in RA and periodontitis. In serum, increased levels of this cytokine have previously been reported in both diseases and seem to correlate with disease activity at least in RA subjects [60,61]. The duration of treatment with RA medication, however, may have different effects (both increase and decrease) on the levels of Chitinase 3-Like-1 in patients with RA. Knudsen et al. showed that the serum levels of the protein is reduced after 4-, 8- and 12 weeks of methotrexate therapy, but not after 16 weeks [62]. Although the levels of serum and salivary cytokines do not always correlate [63], one explanation for the decreased levels of Chitinase 3-Like-1, observed in our study, might be due to potential differences in the duration of methotrexate therapy between the groups. Unfortunately, we were not able to investigate this due to lack of information about the duration of different RA therapies.

In this study, we did not observe any association between periodontitis severity and RA disease activity in terms of DAS28, the self-assessed health (HAQ-score) or in the levels of CRP (serum, saliva or GCF). In agreement with these findings, several studies report no impact of the severity of periodontitis on RA disease activity or on RA-associated serological markers [64,65,66]. For example, in a cohort of 100 RA patients and 112 matched controls, no association was found between RA disease activity (assessed by DAS28 score) and periodontitis severity [65]. In contrast, some studies have detected an association between periodontitis/alveolar bone loss and measurements of RA disease activity/severity such as DAS28, CRP, HAQ, tender joint count and/or joint space narrowing scores [42,67,68,69]. Three of these studies, conducted in the same cohort of 287 RA subjects, reported an association between periodontitis diagnosis/alveolar bone loss or self-reported “loose teeth” and higher DAS28-/HAQ-score and tender/swollen joint count [42,68,69]. The inconsistent results from different studies and the lack of association between periodontitis and RA may be due to RA medication. For example, DMARDs and biological DMARDs could be confounding factors potentially masking an association by decreasing RA disease activity or periodontitis severity [70,71,72,73]. In our study, however, there were no differences in type of medication used based on periodontitis, suggesting that the type of medication alone may not explain the lack of association between periodontitis and RA disease activity in this study. It is, however, plausible that the patients may respond differently to RA medication, or that different subtypes of DMARDs/biological DMARDs may affect periodontitis differently. In addition, the contrasting results between the different studies may also be due to the lack of uniformity in defining periodontitis [74].

One of the strengths of the current study is the well-characterised RA cohort and data set, including information on several potential confounding factors for RA and periodontitis, such as RA disease duration, BMI, comorbidities, type of RA medication used, smoking, alcohol consumption and education. In addition, the gender distribution also reflected the general RA population as the majority of the participants were women [75]. Furthermore, despite the limited number of patients in this pilot study (especially in the no/mild periodontitis group), our results still indicate that cigarette-exposure is associated with the ACPA-positive RA subset as well as with the severity of periodontitis, in agreement with previous findings [76,77]. A limitation of this study is the higher age of the patients with moderate/severe periodontitis compared to no/mild disease. Since age is known to be strongly correlated with periodontitis disease severity [76], this might potentially affect the results. On the other hand, no differences in age were detected between the groups based on ACPA status, showing an increased frequency of ACPA positivity among patients with moderate/severe periodontitis. Nevertheless, the results should be interpreted with caution due to limitations of the study including small samples size and the lack of information about the duration of different RA medications. Furthermore, data about the levels of ACPA and RF were lacking, and we have instead used a positive/negative antibody classification following the ACR classifications criteria for RA. Additional studies using the levels of antibodies, particularly the fine specificities of anti-CCP/ACPA antibodies, in relation to the disease outcome could be relevant.

## 5. Conclusions

In conclusion, our findings demonstrate that patients with ACPA-positive RA have more severe forms of periodontitis, irrespective of DMARD therapy or the presence of subgingival *P. gingivalis*. Moreover, our data show a different subgingival microbial profile in RA patients with moderate/severe periodontitis *versus* no/mild periodontal disease. In addition, we also report, to our knowledge for the first time, that RA subjects with moderate/severe periodontitis have increased serum and salivary levels of the proliferation-inducing ligand APRIL. This cytokine, known to be important for B-cell survival and maturation, could potentially be involved in the association between RA and periodontitis, although additional studies including larger number of patients are required for confirmation of this finding.

## Figures and Tables

**Table 1:** Characteristics of subjects with rheumatoid arthritis (RA) in relation to periodontal diagnosis.

Characteristics	No/Mild Periodontitis (*n* = 10)	Moderate/Severe Periodontitis (*n* = 30)	*p* Value
Gender, no (%)			
Female	10 (100)	25 (83)	
Male	0 (0)	5 (17)	0.306
**Age, mean (±SD)**	50 (14)	64 (7.8)	0.004
**BMI, mean (±SD)**	26 (6.8)	24 (5.6)	0.325
**RA duration in years, mean (±SD)**	12 (11)	10 (10)	0.406
**Comorbidities, no (%)**			
Diabetes	0 (0)	2 (6.7)	1.000
Cardiovascular disease	3 (30)	8 (27)	1.000
High blood pressure	2 (20)	5 (17)	1.000
Gastrointestinal disorders	1 (10)	6 (20)	0.739
Osteoporosis	0 (0)	3 (11)	0.552
Asthma	0 (0)	5 (17)	0.306
Sjögren’s syndrome	0 (0)	2 (7.4)	1.000
TMJ	4 (40)	8 (27)	0.451
**Medication, no (%)**			
Analgesics	6 (60)	13 (43)	0.473
NSAIDs	1 (10)	10 (33)	0.233
DMARDs	6 (60)	22 (73)	0.451
Biological DMARDs	5 (50)	11 (37)	0.482
Glucocorticoids	5 (50)	18 (60)	0.717
Bisphosphonates	1 (10)	1 (3.0)	0.442
**Smoking habits, no (%)**			
Current smokers	0 (0)	5 (17)	0.306
Ex-smokers	4 (40)	19 (73)	0.119
Never smokers	6 (60)	5 (17)	**0.014**
**Alcohol consumption, no (%)**			
Monthly	7 (70)	20 (67)	1.000
Weekly	5 (50)	14 (47)	1.000
Daily	0 (0)	3 (10)	0.560
Never	2 (20)	3(10)	0.584
**Education, no (%)**			
University degree	4 (40)	13 (46)	
No university degree	6 (60)	5 (54)	1.000
**Place of birth, no (%)**			
Sweden	10 (100)	24 (83)	
Other	0 (0)	5 (17)	0.302

RA, rheumatoid arthritis; BMI, body mass index; TMJ, disorders involving the temporomandibular joint; NSAIDs, non-steroidal anti-inflammatory drugs; DMARDs, disease-modifying antirheumatic drugs; SD, standard deviation. Differences between the groups were analysed by Chi-square test or Fisher´s exact test for categorical variables, and Mann–Whitney U-test continuous demographics. *p* value < 0.05 was considered statistically significant.

**Table 2:** Rheumatological and serological characteristics in relation to periodontal diagnosis.

Characteristics	No/Mild Periodontitis (*n* = 10)	Moderate/Severe Periodontitis (*n* = 30)	*p* Value
**ACPA status, no (%)**			
ACPA-positive	5 (50)	25 (86)	
ACPA-negative	5 (50)	4 (14)	0.032
**RF status, no (%)**			
RF-positive	5 (50)	22 (73)	
RF-negative	5 (50)	8 (27)	0.246
**HAQ score (range 0–3), mean (±SD)**	0.7 (0.5)	0.9 (0.6)	0.380
**DAS28 score (range 1-10), mean (±SD)**	2.7 (1.5)	3.3 (1.4)	0.411
**Disease activity (DAS28 score), no (%)**			
Remission (score < 2.6)	4 (44)	10 (37)	1.000
Low activity (score 2.6–3.2)	0 (0)	4 (15)	0.553
Moderate activity (score 3.2–5.1)	5 (56)	9 (33)	0.432
High activity (score >5.1)	0 (0)	4 (15)	0.553
**Serum CRP (ng/mL), mean (±SD)**	5100 (7300)	4300 (5200)	0.353
**Salivary CRP (ng/mL), mean (±SD)**	1.4 (1.9)	2.1 (5.4)	0.749
**GCF CRP (ng/mL), mean (±SD)**	2.2 (3.6)	2.3 (5.4)	0.617

ACPA, anti-citrullinated protein antibodies; RF, rheumatoid factor; HAQ, health assessment questionnaire; DAS28, 28-joint disease activity score; CRP, C-reactive protein; GCF, gingival crevicular fluid; SD, standard deviation. Differences between the groups were analysed by chi-square test or Fisher´s exact test for categorical variables, and Mann–Whitney U-test for ordinal or continuous demographics. *p* value < 0.05 was considered statistically significant.

**Table 3:** Levels of inflammatory mediators (mean ±SD) and total protein (mg/mL) in gingival crevicular fluid (GCF) of subjects with RA in relation to periodontal diagnosis.

Variables	No/Mild Periodontitis (*n* = 9)	Moderate/Severe Periodontitis (*n* = 29)	*p* Value
APRIL (TNFSF13)	5289 (2451)	4584 (935)	0.652
BAFF (TNFSF13B)	2229 (736)	2982 (1767)	0.440
sCD30 (TNFRSF8)	9.0 (11)	21 (18)	0.077
sCD163	2980 (2727)	3678 (2835)	0.400
Chitinase 3-like 1	7284 (3881)	12,885 (9084)	0.118
gp130 (sIL-6Rß)	585 (424)	713 (828)	0.823
IFN-α2	20 (27)	43 (31)	0.036
sIL-6Rα	456 (339)	786 (594)	0.175
IL-8	761 (372)	1274 (995)	0.345
IL-10	2.0 (1.6)	4.3 (5.5)	0.104
IL-11	1.9 (2.1)	2.6 (4.4)	0.692
IL-12 (p70)	2.9 (3.2)	3.8 (4.4)	0.635
IL-19	14 (13)	32 (37)	0.035
IL-20	68 (51)	76 (26)	0.718
IL-22	6.6 (4.5)	10 (9.1)	0.353
IL-26	15 (11)	36 (62)	0.046
IL-27 (p28)	21 (11)	21 (10)	0.810
IL-29 (IFN-γ1)	52 (61)	103 (186)	0.198
IL-32	103 (55)	140 (92)	0.319
IL-34	205 (127)	184 (101)	0.436
LIGHT (TNFSF14)	269 (93)	314 (117)	0.328
MMP-1	897 (636)	1294 (534)	0.049
MMP-3	394 (298)	489 (371)	0.579
Pentraxin-3	375 (261)	429 (272)	0.718
sTNF-R1	259 (130)	523 (361)	0.015
sTNF-R2	2075 (5013)	1976 (5322)	0.311
TSLP	24 (20)	25 (15)	0.796
TWEAK (TNFSF12)	27 (17)	38 (16)	0.074
Total protein concentration	0.2 (0.2)	0.3 (0.3)	0.503

GCF, gingival crevicular fluid; RA, rheumatoid arthritis; SD, standard deviation. Differences between the groups were analysed by Mann–Whitney U-test. *p* value < 0.05 was considered statistically significant.

## Supplementary Materials

The following are available online at https://www.mdpi.com/2077-0383/8/5/630/s1, Table S1: Periodontal and dental characteristics in relation to periodontal diagnosis, Table S2: Characteristics of subjects with RA in relation to the ACPA status, Table S3: Most abundant bacteria in saliva of patients with RA in relation to the periodontal diagnosis, Table S4. Presence of subgingival *P. gingivalis* in relation to the periodontal diagnosis. Supplementary Methods.
