# Supplementary material for: Periodontal Health and Oral Microbiota in Patients with Rheumatoid Arthritis
Source: J Clin Med. 2019 May 8;8(5):630. doi: 10.3390/jcm8050630 (PMC6572048; doi:10.3390/jcm8050630)
Supplement: Supplementary file 1 [file jcm-08-00630-s001.pdf]

**Supplementary table S1**. Periodontal and dental characteristics in relation to periodontal diagnosis.

| Characteristics | No/Mild Periodontitis (*n* = 10) | Moderate/Severe Periodontitis (*n* = 30) | *p* Value |
|---|---|---|---|
| **Plaque index, mean % (±SD)** | 49 (8.9) | 48 (21) | 0.926 |
| **Bleeding index, mean % (±SD)** | 32 (18) | 34 (21) | 0.781 |
| **Sites with PPD, mean no (%)** | | | |
| ≥4 mm | 2.3 (1.4) | 14 (9.5) | **<0.001** |
| **Interproximal sites with PPD ≥5 mm, mean no (±SD)** | 0 (0) | 2.4 (2.6) | **<0.001** |
| **Sites with CAL, mean no (%)** | | | |
| 1-2 mm | 112 (70) | 54 (37) | **<0.001** |
| 3-4 mm | 39 (24) | 73 (49) | **0.005** |
| ≥5 mm | 1.0 (0.6) | 17 (12) | **0.001** |
| **Interproximal sites with CAL, mean no (±SD)** | | | |
| ≥4 mm | 0.5 (0.5) | 10 (6.7) | **<0.001** |
| ≥6 mm | 0 (0) | 2 (3.4) | **0.003** |
| **Missing teeth, mean no (±SD)** | 1.0 (1.4) | 3.5 (3.5) | **0.003** |
| **Mobile teeth, mean no (±SD)** | 0.1 (0.3) | 1.5 (2.6) | **0.006** |
| **Furcation involvement, mean no (±SD)** | 1.7 (2.3) | 3.9 (2.3) | **0.015** |
| **DMFT, mean (±SD)** | 14 (5.9) | 21 (8.0) | **0.021** |
| **DMFS, mean (±SD)** | 35 (23) | 59 (27) | **0.022** |
| **Stimulated salivary flow rate (ml/min), mean (±SD)** | 1.5 (0.5) | 1.7 (1.0) | 0.628 |

PPD, probing pocket depth; CAL, clinical attachment level; DMFT, decayed, missing and filled teeth; DMFT, decayed, missing and filled surfaces; SD, standard deviation. Differences between the groups for continuous demographics were analyzed by Mann-Whitney U-test. *p* value < 0.05 was considered statistically significant.

**Supplementary Table S2.** Characteristics of subjects with RA in relation to the ACPA status.

| Characteristics | ACPA-Negative (*n* = 9) | ACPA-Positive (*n* = 30) | *p* Value |
|---|---|---|---|
| **Gender, no (%)** | | | |
| Female | 8 (89) | 26 (87) | |
| Male | 1 (11) | 4 (13) | 1.000 |
| **Age, mean (±SD)** | 52 (18) | 62 (7.4) | 0.802 |
| **BMI, mean (±SD)** | 25 (7.1) | 25 (5.4) | 0.526 |
| **RA duration in years, mean (±SD)** | 12 (17) | 9.6 (7.2) | 0.432 |
| **Comorbidities, no (%)** | | | |
| Diabetes | 0 (0) | 2 (6.7) | 1.000 |
| Cardiovascular disease | 2(22) | 9 (30) | 1.000 |
| High blood pressure | 1 (11) | 5 (17) | 1.000 |
| Gastrointestinal disorders | 0 (0) | 7 (23) | 0.357 |
| Osteoporosis | 0 (0) | 3 (10) | 1.000 |
| Asthma | 0 (0) | 4 (13) | 0.556 |
| Sjögren's syndrome | 0 (0) | 2 (7.1) | 1.000 |
| TMJ | 2 (22) | 3 (33) | 0.693 |
| **Medication, no (%)** | | | |
| Analgesics | 3 (33) | 15 (50) | 0.464 |
| NSAIDs | 0 (0) | 11 (37) | **0.040** |
| DMARDs | 6 (67) | 21 (70) | 1.000 |
| Biological DMARDs | 3 (33) | 13 (43) | 0.711 |
| Glucocorticoids | 7 (78) | 15 (50) | 0.251 |
| Bisphosphonates | 0 (0) | 2 (6.7) | 1.000 |
| **Smoking habits, no (%)** | | | |
| Current smokers | 1 (11) | 4 (13) | 1.000 |
| Ex-smokers | 3 (33) | 20 (77) | **0.038** |
| Never smokers | 5 (56) | 5 (17) | **0.032** |
| **Alcohol consumption, no (%)** | | | |
| Monthly | 7 (78) | 19 (63) | 0.689 |
| Weekly | 4 (44) | 15 (50) | 1.000 |
| Daily | 0 (0) | 3 (10) | 1.000 |
| Never | 0 (0) | 5 (17) | 0.318 |
| **Education, no (%)** | | | |
| University degree | 2 (22) | 15 (52) | |
| No university degree | 7 (78) | 14 (58) | 0.148 |
| **Place of birth, no (%)** | | | |
| Sweden | 8 (89) | 26 (87) | |
| Other | 1 (11) | 4 (13) | 1.000 |

RA, rheumatoid arthritis; ACPA, anti-citrullinated protein antibodies; BMI, body mass index; TMJ, disorders involving the temporomandibular joint; NSAIDs, non-steroidal anti-inflammatory drugs; DMARDs, disease-modifying anti-rheumatic drugs; SD, standard deviation. The differences between the groups were analyzed by chi-square test or Fisher´s exact test for categorical variables, and Mann-Whitney U-test for continuous demographics. *p* value $< 0.05$ was considered statistically significant.

**Supplementary Table S3**. Most abundant bacteria in saliva of patients with RA in relation to the periodontal diagnosis.

| | Genus | Species | Median Abundance (%) |
|---|---|---|---|
| **No/Mild Periodontitis (*n* = 10)** | | | |
| | *Streptococcus* | *dentisani/infantis/mitis/oligofermentans/oralis/pneumoniae/pseudopneumoniae/sanguinis/tigurinus* | 2.70 |
| | *Haemophilus* | *parainfluenzae* | 0.59 |
| | *Veillonella* | *dispar/parvula* | 0.52 |
| | *Granulicatella* | *adiacens/para-adiacens* | 0.50 |
| | *Streptococcus* | *mitis/parasanguinis* | 0.46 |
| | *Prevotella* | *melaninogenica* | 0.41 |
| | *Streptococcus* | *sanguinis* | 0.34 |
| | *Gemella* | *haemolysans/sanguinis* | 0.31 |
| | *Fusobacterium* | *nucleatum/periodonticum* | 0.28 |
| | *Campylobacter* | *concisus* | 0.24 |
| **Moderate/Severe Periodontitis (*n* = 28)** | | | |
| | *Streptococcus* | *dentisani/infantis/mitis/oligofermentans/oralis/pneumoniae/pseudopneumoniae/sanguinis/tigurinus* | 4.69 |
| | *Veillonella* | *dispar* | 0.82 |
| | *Haemophilus* | *parainfluenzae* | 0.57 |
| | *Rothia* | *dentocariosa* | 0.46 |
| | *Granulicatella* | *adiacens/para-adiacens* | 0.45 |
| | *Prevotella* | *melaninogenica* | 0.39 |
| | *Veillonella* | *dispar/parvula* | 0.31 |
| | *Streptococcus* | *salivarius/vestibularis* | 0.21 |
| | *Streptococcus* | *sanguinis* | 0.20 |
| | *Fusobacterium* | *canifelinum/nucleatum* | 0.20 |

**Supplementary Table S4**. Presence of subgingival *P. gingivalis* in relation to the periodontal diagnosis.

| Characteristics | No/Mild Periodontitis ($n$ = 6) | Moderate/Severe Periodontitis ($n$ = 24) | $p$ Value |
|---|---|---|---|
| **Patients with *P. gingivalis*, no (%)** | | | |
| Positive | 3 (50) | 15 (62) | |
| Negative | 3 (50) | 9 (38) | 0.660 |

*P. gingivalis*, *Porphyromonas gingivalis*. Differences between the groups were analyzed by Fisher´s exact test. $p$ value < 0.05 was considered statistically significant.

**Supplementary Methods**

*Preparation of Samples*

Each of the collected GCF and subgingival plaque samples, consisting of two paper strips/GCF sample and four paper points/plaque sample, were diluted in 200 µl of PBS (pH7.4) with 0.05% Tween-20 (for GCF) or 200 µl of PBS (for plaque). Samples were vortexed for 30 s and stored thereafter at -80˚C until analysis. The saliva samples (400 µl/sample) were centrifuged at 10,000 rpm for 15 min, and the pellets resuspended in 200 µl of PBS, prior to DNA extraction. Total protein concentration was assessed in each of the GCF and saliva samples by using the DC™ (detergent compatible) protein assay (Bio-Rad, Hercules, CA, USA) following the instructions of the manufacturer. A serial dilution of bovine serum albumin (Sigma-Aldrich, St. Louis, Missouri, USA) was used to prepare the standard curve. Absorbance reading was measured at 690 nm with a microplate reader (Multiskan MS Type 352, Labsystems, Finland). Bacterial DNA was isolated from plaque samples by using a DNA extraction kit (QiaAmp DNA Mini Kit, QIAGEN, Sweden) according to manufacturer´s recommendations. In brief, the samples were resuspended in tissue lysis buffer and lysed using proteinase K (QIAGEN) at 56ºC (10 min for saliva and 30 min for plaque), purified using ethanol-containing buffers, and eluted in nuclease-free water, according to manufacturer's protocol. The amount of DNA was quantified by Qubit™ 2.0 fluorometer (Invitrogen, Life Technologies, USA).

*Quantitative Polymerase Chain Reaction, qPCR*

The qPCR was performed using a total reaction volume of 20 µl containing 5 ng of the gDNA sample, TaqMan Universal PCR Master Mix (1X) (Applied Biosystems, USA), the specific primers at a final concentration of 0.9 µM each and the probe of 0.25 µM (Eurofins Genomics, Ebersberg, Germany). All samples were analyzed in duplicates. The qPCR was programmed as follows, initial incubation for 2 min at 50°C, followed by denaturation for 10 min at 95°C and 40 PCR cycles (95°C for 15 s, 60 °C for 1 min). The previously described [1,2] primers and probe for *P. gingivalis* were forward: 5′-GCGCTCAACGTTCAGCC-3′, reverse: 5′-CACGAATTCCGCCTGC-3′ and probe: 5′-[6FAM]–CACTGAACTCAAGCCCGGCAGTTTCAA–[TAMRA]-3′. For 16S rRNA gene the primers were forward: 5′-TGGAGCATGTGGTTTAATTCG A-3′, reverse: 5′-TGCGGGACTTAACCCAACA-3′ and probe: 5′-[6FAM]–CACGAGCTGACGACARCCATGCA–[TAMRA]-3′ [1,2].

1. Nonnenmacher, C.; Dalpke, A.; Mutters, R.; Heeg, K. Quantitative detection of periodontopathogens by real-time PCR. *J Microbiol Methods*. **2004**, *59*, 117-125.
2. Eriksson, K.; Lonnblom, E.; Tour, G.; Kats, A.; Mydel, P.; Georgsson, P.; Hultgren, C.; Kharlamova, N.; Norin, U.; Jonsson, J., et al. Effects by periodontitis on pristane-induced arthritis in rats. *J Transl Med*. **2016**, *14*, 311.
